# SWiLoc: Fusing Smartphone Sensors and WiFi CSI for Accurate Indoor Localization

**DOI:** 10.3390/s24196327

**Published:** 2024-09-30

**Authors:** Khairul Mottakin, Kiran Davuluri, Mark Allison, Zheng Song

**Affiliations:** 1 Department of Computer and Information Science, University of Michigan-Dearborn, Dearborn, MI 48128, USA; khairulm@umich.edu (K.M.); kirandav@umich.edu (K.D.); 2 College of Innovation and Technology, University of Michigan-Flint, Flint, MI 48502, USA; markalli@umich.edu

**Keywords:** Channel State Information (CSI), dead reckoning, indoor localization, smartphone sensor fusion, walking direction estimation, WiFi sensing

## Abstract

Dead reckoning is a promising yet often overlooked smartphone-based indoor localization technology that relies on phone-mounted sensors for counting steps and estimating walking directions, without the need for extensive sensor or landmark deployment. However, misalignment between the phone’s direction and the user’s actual movement direction can lead to unreliable direction estimates and inaccurate location tracking. To address this issue, this paper introduces SWiLoc (**S**martphone and **Wi**Fi-based **Loc**alization), an enhanced direction correction system that integrates passive WiFi sensing with smartphone-based sensing to form Correction Zones. Our two-phase approach accurately measures the user’s walking directions when passing through a Correction Zone and further refines successive direction estimates outside the zones, enabling continuous and reliable tracking. In addition to direction correction, SWiLoc extends its capabilities by incorporating a localization technique that leverages corrected directions to achieve precise user localization. This extension significantly enhances the system’s applicability for high-accuracy localization tasks. Additionally, our innovative Fresnel zone-based approach, which utilizes unique hardware configurations and a fundamental geometric model, ensures accurate and robust direction estimation, even in scenarios with unreliable walking directions. We evaluate SWiLoc across two real-world environments, assessing its performance under varying conditions such as environmental changes, phone orientations, walking directions, and distances. Our comprehensive experiments demonstrate that SWiLoc achieves an average 75th percentile error of 8.89 degrees in walking direction estimation and an 80th percentile error of 1.12 m in location estimation. These figures represent reductions of 64% and 49%, respectively for direction and location estimation error, over existing state-of-the-art approaches.

## 1. Introduction

Accurately determining user location can facilitate numerous applications such as navigation, social media, and location-based marketing [1]. While GPS is the preferred method for outdoor localization, its effectiveness for indoors is restricted due to signal degradation or blockage by physical obstacles. Beyond GPS, indoor localization techniques fall into two categories: device-free and smartphone-based. However, these methods are not widely adopted in pervasive indoor settings due to their inherent constraints [2].

Smartphones have become indispensable in our daily activities and are expected to remain popular in the years to come. Technologies for indoor localization based on smartphones include WiFi RSSI [3], Pedestrian Dead Reckoning (PDR) [4], BLE-beacon [5], and camera-based methods [6]. Among these, PDR is particularly practical for widespread use as it utilizes the IMU sensors built into smartphones. While distance measurement via IMU sensors is precise, estimating walking direction is less reliable due to the misalignment between the smartphone’s orientation and the actual direction of human movement. Accurate direction estimation is crucial as it significantly enhances the ability to estimate a user’s accurate location within complex indoor environments, thus improving the overall effectiveness of navigation and location-dependent applications. This directional misalignment can lead to significant errors in location determination [7].

Building on previous discussions regarding smartphone-based localization technologies, considerable research has been directed toward improving the accuracy of direction estimation using smartphones. These methodologies generally fall into two categories: those that depend on external landmarks [8,9,10] and those that enhance IMU sensor data through various statistical techniques [11,12,13,14,15]. The former requires users to consistently pass by designated landmarks, which may not always be feasible in fluid urban environments. The latter, while effective, tends to be tailored to individual users, reflecting specific gait patterns and how the device is carried, which can limit its general applicability and sensitivity.

In this paper, we improve Pedestrian Dead Reckoning (PDR)-based direction estimation by integrating it with passive WiFi sensing to enhance overall localization accuracy. Passive WiFi sensing, a widely studied device-free indoor localization method, leverages the ubiquitous presence of WiFi. By merging the benefits of smartphone-based motion sensing with WiFi sensing, we establish WiFi sensing regions (correction zones) using WiFi devices. We then utilize the precise walking distances recorded by smartphones to refine the accuracy of WiFi sensing. This integrated approach not only yields superior accuracy and reliability compared to standalone WiFi-based direction estimation but also effectively enhances the precision of location estimation, addressing the limitations inherent in smartphone-based direction estimation methodologies.

This paper is an extended version of our previous work [16], which was presented as a short paper at the MASS’23. In this version, we expanded the methodology by establishing a correlation between the phone’s compass direction and the user’s walking direction (we call it Phase 1); as well as by using this correlation to estimate the user’s walking direction based on the phone’s compass direction (Phase 2). We used this accurate walking direction to estimate user’s precise location. Additionally, we extended the experimental results by conducting real-world experiments in continuous traces. Finally, we provided a more comprehensive analysis on how different phone holding positions impacts the walking direction accuracy, thereby the localization accuracy. We also compared these findings with state-of-the-art localization approaches.

In summary, our main contributions are as follows:

- In this paper, we introduce SWiLoc (**S**martphone and **Wi**Fi-based **Loc**alization), a novel direction correction system that leverages passive WiFi sensing to form Correction Zones for refining smartphone-based user direction estimates. Our **two-phase** approach not only accurately measures the user’s walking directions when they pass through a correction zone but also utilizes these measured directions to estimate their successive directions outside correction zones. This is done by first establishing a correlation in Phase 1 and using this correlation in Phase 2.
- Building on the first contribution, we extend SWiLoc’s capabilities by implementing an accurate localization technique that uses the corrected directions to achieve precise user localization. This extension enhances the system’s utility by enabling continuous and accurate tracking of the user’s movements, providing a robust solution for applications requiring high localization accuracy.
- Our third contribution is the resolution of **unreliable walking directions** through our innovative and distinctive hardware configurations. We discuss and resolve the unreliable direction problem in this paper. Our model is based on the Fresnel zone-based approach that not only ensures reliable direction estimations in challenging scenarios but also significantly enhances localization accuracy.
- Our system undergoes rigorous analysis and evaluation across two real-world settings, where its performance is benchmarked against state-of-the-art methods. We thoroughly assess how various factors—such as environmental conditions, ways the phone is held, walking directions, and varying locations and distances—affect the precision of our method. The results demonstrate that SWiLoc consistently outperforms other existing methods in both direction estimation and localization, regardless of whether they utilize WiFi sensing or smartphone sensor fusion.

The structure of this paper is organized as follows: Section 2 reviews existing solutions and identifies their limitations. Section 3 describes our system’s overview and design principles. Section 4 details the methodology of our system. Section 5 offers a comprehensive description of our system’s implementation. The evaluation results are presented in Section 6. The paper concludes with Section 7.

## 2. Related Works and Background

Numerous studies have focused on precisely determining the walking direction and location of an individual using either smartphone IMU sensors or WiFi CSI (Channel State Information) [17,18]. Each method has its inherent drawbacks. In this section, we provide a concise overview of several recent and notable studies, highlighting their advantages and disadvantages.

### 2.1. Channel State Information (CSI)

CSI is extensively utilized to analyze the propagation dynamics of WiFi signals when encountering physical barriers [19]. WiFi technologies typically employ OFDM modulation, which spreads the signal over multiple subcarriers. Unlike the Received Signal Strength Indicator (RSSI), which averages signal strengths across subcarriers, CSI detects individual subcarrier fluctuations, offering detailed measurements. CSI data is structured as a three-dimensional channel tensor involving *t* transmitting and *r* receiving antennas:

$$CSI=\left[\begin{array}{ccc} H_{1,1} & \cdots & H_{1,r}\\ \vdots & \ddots & \vdots \\ H_{t,1} & \cdots & H_{t,r} \end{array}\right]$$

Here, $H_{t,r}$ is a vector comprising complex pairs for each sub-carrier:

$$H_{t,r}=\left[h_{t,r,1},\cdots ,h_{t,r,m}\right]$$

The count of subcarriers varies with the hardware and bandwidth used. Each subcarrier in $H_{t,r}$ is represented as a complex number $h_{m}$, incorporating both amplitude ($|h_{m}|$) and phase ($∠h_{m}$) components. Multipath effects like phase shifts and amplitude attenuation impact these CSI values, which are crucial for precisely detecting human movements and locations.

### 2.2. Pedestrian Dead Reckoning (PDR)

Dead reckoning involves continuously calculating the distance or direction from a fixed starting point to determine the current location in indoor navigation settings. In PDR, the number of steps and the length of each step are utilized to calculate the distance a pedestrian has covered, while direction estimation helps to find the pedestrian’s current heading. Understanding both the direction and the step length enables pedestrians to find their real-time positions from an initial location.

The primary methods for counting steps through a smartphone’s built-in accelerometer include threshold setting, peak detection, correlation analysis, and spectral analysis, as noted in [20]. Research indicates that these existing step counting methods are highly accurate, with [20] reporting error rates below 5% for the majority of pedestrians.

Numerous step length estimation algorithms necessitate customized training for each user, as discussed in [21]. However, recent advancements have led to the development of several training-free methods [22] that accurately determine step length regardless of the individual pedestrian. In this paper, we implemented a simple yet accurate step count and step length estimation method, although our main focus is to accurately identify pedestrian’s heading direction and location.

The compass is the standard method for estimating direction embedded in both Android and iOS devices. However, the discrepancy between the orientation of the phone and the actual walking direction of the user results in only moderate accuracy when using a smartphone compass for dead reckoning, as noted in [7]. Following this, we will examine and encapsulate the latest advancements in walking direction and location estimation techniques.

### 2.3. Smartphone Sensor Fusion Based Direction and Location Estimation

By sensor fusion, only using the sensor readings obtained from a smartphone (accelerometer, gyroscope, and magnetometer) can get a precise estimation of users’ walking direction [7,11,12,13,14,15,23,24,25]. These approaches calculate the cumulative acceleration of pedestrian and project it to the horizontal plane, by associating the 3D angular rotation of the phone (captured by gyroscope) with 3D accelerometer and magnetometer readings. We further divide these approaches into two sub-categories, based on whether they require user-specific training or not.

**Training-based:** The training-based approaches [11,12,13,14,15,23] obtain context information on human motion and device attachment to more accurately estimate their directions. To do so, they employ statistical techniques (Naive Bayes, Kalman Filter or Particle Filter), classifiers or Machine Learning models(CNN, LSTM etc.) However, these methods require labor-intensive data collection. Moreover, one specific threshold value might not work for different pedestrians, causing negative impact on accuracy. Lastly, these approaches typically demand pedestrians to walk for a long distance, thus create lag while inferring direction.

**Training-free:** Some training-free approaches (e.g., SmartPDR [24] and RMPCA [25]) assume that the pedestrian carries the phone in a fixed mode. They use principle component analysis (PCA) to filter out the noises in direction estimation. Some other approaches (e.g., Humaine [7]) can adapt to multiple mode of users holding the phone. However, all these approaches require certain patterns in user’s walking and holding the phone, which results in low direction estimation accuracy when the requirements are not met.

### 2.4. Calibration-Based Direction and Location Estimation

Calibration-based direction estimation necessitates that the pedestrian traverse between two predefined landmarks. The trajectory of the pedestrian, captured while moving between these landmarks, is monitored, though the phone’s compass may provide unreliable direction. Upon reaching the second landmark, the true walking direction is computed and used to adjust the correlation between the phone’s compass output and the pedestrian’s actual movement direction. These landmarks can include options such as LED lights [9], acoustic markers [10], or Bluetooth beacons [26,27].

Nevertheless, calibration-based direction estimation methods face several limitations: (1) they often necessitate the installation of additional hardware devices; (2) their effectiveness is limited by environmental conditions (for instance, SoundMark is ineffective in noisy settings and LiDR requires LED lights to be arranged in a specific configuration); (3) they require that pedestrians travel through two sequential locations, which introduces an additional complication.

### 2.5. Direction and Location Estimation Using WiFi

Device-free direction and location estimation is a method for determining the movement direction and location of a pedestrian without any devices carried by the individual. This approach utilizes existing wireless signals, such as WiFi, within the environment. These signals reflect off the moving pedestrian, and the resulting reflections are captured by several receivers positioned throughout the area. Research such as WiDir [28], WiDar [29] and WiDar2 [30] determines the user’s direction using device-free techniques by implementing various theoretical models, including the Fresnel zone, Doppler Frequency Shift (DFS), Time-of-Flight (ToF), and Angle-of-Arrival (AoA). Notably, the Fresnel zone model is distinguished by its simple geometrical features and its capability to provide accurate direction and subsequent location estimations.

**Figure 1:**
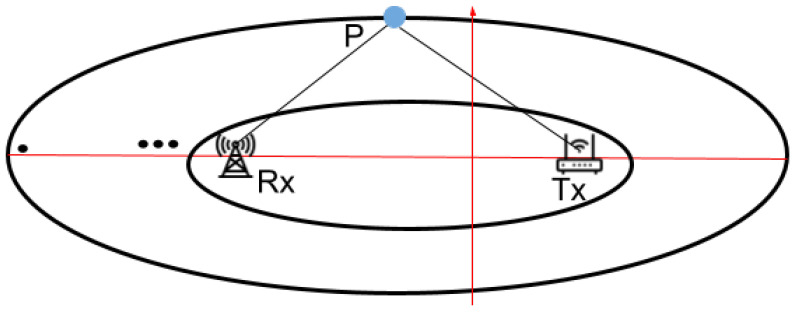
Geometry of Fresnel Zones.

The *Fresnel zone* is created when radio waves travel from a transmitter (Tx) to a receiver (Rx), traversing multiple paths that result in both constructive and destructive interference due to the alternating lengths of these paths [28]. When considering a radio wavelength λ, Fresnel zones consist of *n* ellipses, defined by the following properties:

(1) |RxP|+|PTx|−|TxRx|=nλ/2

where *P* represents a point on the *n*th ellipse, as depicted in Figure 1. The path length (|RxP| + |PTx|) of the signal that is reflected or diffracted through the *n*th Fresnel zone boundary is nλ/2 longer than the direct Line-of-Sight (LoS) path length (|TxRx|). Although an infinite number of Fresnel zones exist, Raspberry Pis are limited to measuring 256 sets of Fresnel zones due to the use of 256 OFDM subcarriers. The innermost ellipsoid is referred to as the First Fresnel Zone (FFZ). Conventional Fresnel Zone approaches exhibit significant errors when users move within certain areas, which will be further explored in Section 3.2.

In summary, the phone compass is inadequate for direction determination in PDR, and each direction estimation method we have explored exhibits specific limitations. We will next provide a design overview of SWiLoc.

## 3. SWiLoc System

In this section, we introduce the SWiLoc system, which integrates passive WiFi sensing with smartphone motion detection to estimate walking direction and location. Subsequently, we will explore the design considerations that shaped the development of the SWiLoc system.

### 3.1. System Overview

**Figure 2:**
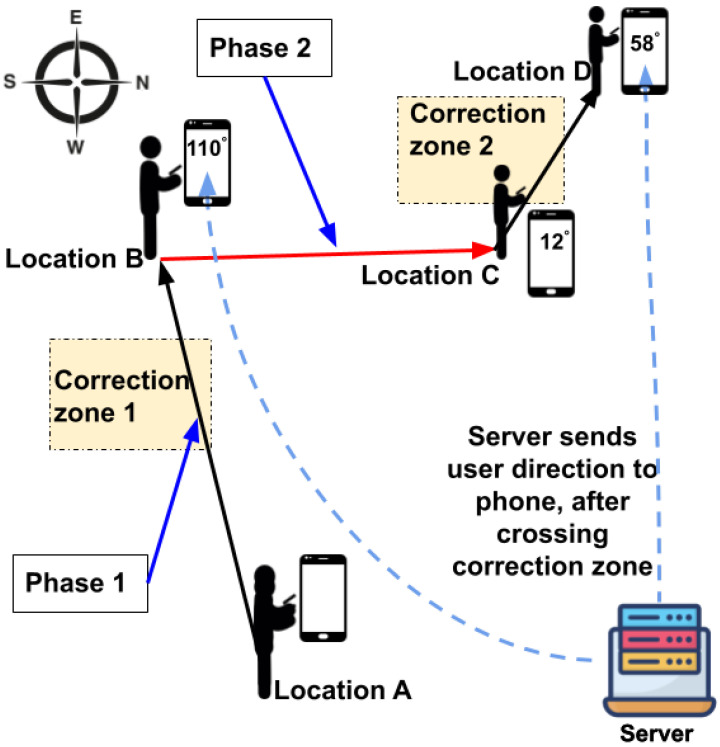
Illustration of SWiLoc System.

SWiLoc, designed to provide walking directions and locations for pedestrians in an indoor space, comprises three primary components: (1) **phones** carried by pedestrians in arbitrary orientation; (2) **correction zones**, each measuring approximately 3 × 3 m and containing a WiFi router placed at center with four WiFi receivers positioned at the corners; (3) a **server** that gathers and processes data from the users’ phones and receivers through wireless links (WiFi or cellular networks), and then transmits the calculated directions back to the phones. Figure 2 illustrates the setup of our system, depicting a user navigating from location A to D via locations B and C, moving through correction zones 1 and 2, with a phone strapped to the their arm.

SWiLoc works **in two phases**: (Phase 1) when the user is crossing the correction zone; (Phase 2) after the user crosses the zone. In phase 1, we utilize the phone’s step count data along with passive WiFi CSI information gathered by WiFi transceivers to determine the user’s walking direction, and correlate their movement direction with the phone’s direction; In phase 2, we collect the phone’s compass direction by which we infer the user’s movement direction and thereby estimate the location using the direction inferred.

To illustrate the functionality of the SWiLoc system, let’s examine a simple scenario depicted in Figure 2. The user begins their journey at point A and concludes at point D, traversing the segments AB, BC, and CD. Upon entering correction zone 1, SWiLoc *phase 1* assesses their walking direction as $110^{\circ }$ relative to global north and communicates this direction to their phone. At point B, the user adjusts their course by $98^{\circ }$ toward global east and proceeds until reaching point C. Since the compass direction diverges from their actual walking trajectory, SWiLoc *phase 2* updates their walking direction using their previous direction and the compass adjustment, resulting in a new direction of $12^{\circ }$.

In summary, SWiLoc consists of two phases. During phase 1, we establish a correlation between the phone’s compass direction and the user’s walking direction. In phase 2, we use this correlation to estimate the user’s walking direction based on the phone’s compass direction. Regardless of how the user holds her phone, SWiLoc can accurately update the real-time walking direction, as long as the phone’s relative position to the user remains unchanged. This allows SWiLoc to function with the phone held in any arbitrary position, such as in the hand palm, shirt pocket, or pant pocket. If the user changes the phone’s orientation while walking, a new correlation will be established when the user passes the next correction zone.

### 3.2. SWiLoc Design Considerations

In most times, SWiLoc users stay in phase 2 and rely on the correlation between phone’s direction and human’s movement direction to infer their directions. The primary challenge for SWiLoc is achieving accurate walking direction and location estimates in phase 1, as phase 2 relies on the results of phase 1. When the user crosses the correction zone, SWiLoc combines the advantages of smartphone-based motion sensing and passive WiFi sensing to improve accuracy.

**Figure 3:**
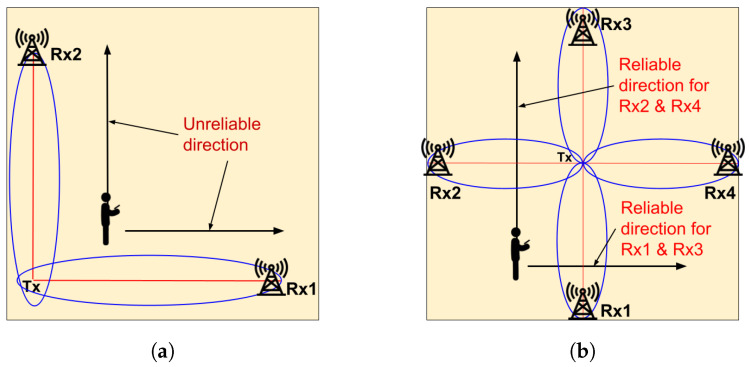
Towards Solving Unreliable Direction Problem. (**a**) Unreliable Direction using 2 receivers. (**b**) SWiLoc solves unreliable direction using 4 receivers.

We opted for the Fresnel zone-based (FZ) method for passive WiFi sensing because it does not require training. Nevertheless, traditional FZ methods are prone to two major issues. Firstly, the FZ model struggles with unreliable directions, which are directions where there is no or very little variation in the CSI data. These directions are parallel or nearly parallel to the Line-of-Sight (LoS) between a transceiver pair as shown in Figure 3a. The direction estimation results become inaccurate when the user walks along these unreliable directions [28]. Secondly, FZ methods estimate direction by calculating the ratio of the number of Fresnel zones traversed by the user along the x and y axes. This approach, being an approximation, introduces errors in the estimation process.

To address the aforementioned problems, SWiLoc introduces innovative solutions, summarized below. The details will be given in the next sections.

(1) To solve the unreliable direction problem, SWiLoc incorporates additional receivers as shown in Figure 3b. As two receivers placed perpendicular to each other may provide inaccurate estimation, we use opposite receivers (either Rx2 and Rx4 or Rx1 and Rx3, depending on the user’s direction) for direction estimation.
(2) In order to adapt to the changes in system hardware setup, SWiLoc utilizes accurate walking distance data from smartphones to improve the precision and reliability of WiFi sensing. Rather than relying on ratios, SWiLoc uses the geometrical relationship between the user’s movement and its effect on the Fresnel zones. This method allows SWiLoc to compute direction and location accurately without resorting to approximations.

## 4. Methodology

This section initially presents the workflow of SWiLoc, followed by comprehensive discussions on the calculation of movement direction (phase 1) and the correction of phone orientation alongside location estimation (phase 2).

### 4.1. Workflow of SWiLoc

**Figure 4:**
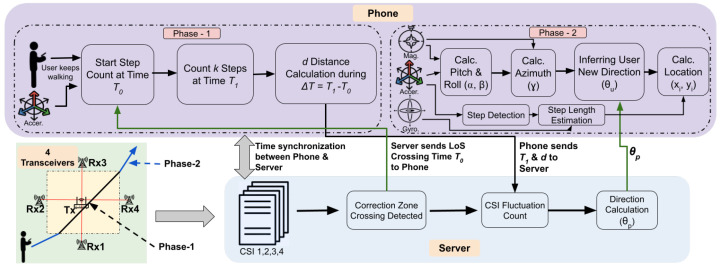
System Workflow of SWiLoc.

Figure 4 illustrates the workflow of SWiLoc. To recap, SWiLoc operates in two phases: WiFi-based direction estimation when the user crosses the correction zone and phone sensor based estimation after the user leaves the zone. The steps and details of both of the phases are described as follows.

**Start:**
  1. The central server integrates a Network Time Protocol (NTP) to ensure time synchronization between the smartphone and all four receivers.
**Phase 1:**
  2. A user with a smartphone enters a correction zone and crosses the Line-of-Sight between a pair of WiFi transceivers at time $T_{0}$. This crossing event is identified through CSI analysis, details of which are elaborated in our system implementation section.
  3. Following CSI analysis, the server transmits $T_{0}$ to the smartphone, which continues to gather data from the motion sensor as the user walks. This data includes the user’s step count, the phone’s orientation (pitch, roll, and azimuth), and the timestamp for each step, all of which are processed and recorded by the smartphone.
  4. The smartphone transmits the time $T_{1}$ and distance *d* to the server, where $T_{1}$ represents the time taken for the user to walk *k* additional steps after crossing the LoS and *d* denotes the distance traveled between $T_{0}$ and $T_{1}$. The value of *k* is predetermined and *d* is calculated using the individual step length of each user.
  5. The server analyzes the CSI data, calculates the fluctuation count between times $T_{0}$ and $T_{1}$ and determines the user’s walking direction $\theta_{p}$ by applying Equations (2) and (3).
  6. The server returns the calculated direction to the smartphone.
**Phase 2:**
  7. The phone receives the user’s walking direction $\theta_{p}$ and maps the phone’s orientation to the user’s walking direction during $T_{0}$ and $T_{1}$ by using Equation (4).
  8. User continues walking, relying on the mapping formed in the previous step to infer the user’s walking direction from phone’s orientation.
  9. Finally, the phone computes user’s location using the corrected walking direction. Phase 1 repeats when the user moves into a next correction zone.

### 4.2. Computation of Direction in Correction Zone

**Figure 5:**
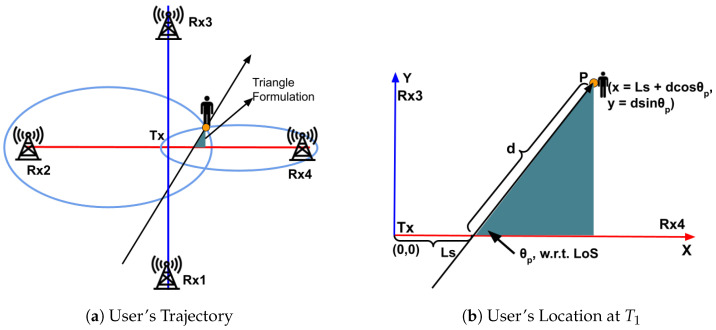
Geometrical Derivation of SWiLoc using Triangle.

Figure 5a illustrates the method for determining the user’s direction using motion sensor data and WiFi sensing. A coordination system is established using a transmitter Tx at (0,0) and four receivers (Rx1–Rx4), with Rx4 aligning with the positive x-axis. When the user intersects the LoS between Tx and Rx4 at time $T_{0}$, the intersection point is designated as (ls,0). Although the CSI analysis at the receiver detects the time of LoS crossing, the exact crossing point, ls, remains undetermined. The user then walks *k* steps to reach point *P* at time $T_{1}$. As detailed in Figure 5b, the coordinates of *P* (user’s location at $T_{1}$) can be represented as:

(2) $$\left(x=ls+d\cos\theta_{p},y=d\sin\theta_{p}\right)$$

Here, ls represents the distance from Tx to the user’s crossing point on the LoS at time $T_{0}$, and $\theta_{p}$ indicates the direction the user is walking from location *P* towards the WiFi transceiver system’s coordinate system. In the formula, the smartphone provides the distance *d* from the LoS crossing to point *P*, with ls and $\theta_{p}$ remaining as the two unknown parameters.

**Figure 6:**
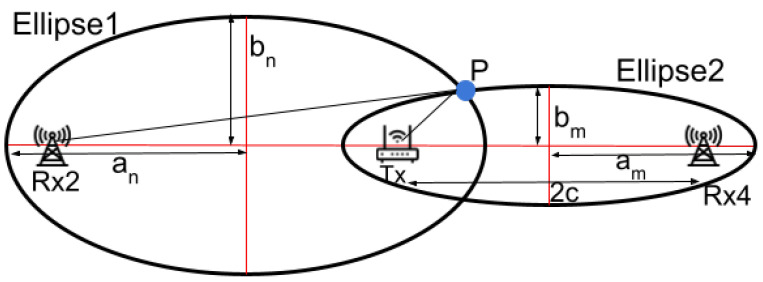
The *m*th and *n*th Fresnel Zone for Rx2 and Rx4.

As the user moves between $T_{0}$ and $T_{1}$, they cross several Fresnel zones established between the pairs of transceivers, Tx and Rx2, and Tx and Rx4. These Fresnel zone crossings are identifiable by analyzing the CSI from the corresponding receivers, which display fluctuations in amplitude levels. For ease of understanding and representation, we simplify the sequence of Fresnel zones at location *P* by counting the number of zones crossed by the user [31]. We assign *m* and *n* to represent the sequence of the Fresnel zones at location *P* for Rx2 and Rx4, respectively, which are determined by counting these fluctuations. Based on the Fresnel zone model, we establish the following:

(3) $$\begin{array}{c} ellipse1:\frac{{\left(x-c\right)}^{2}}{{\left(a_{1}^{n}\right)}^{2}}+\frac{{\left(y\right)}^{2}}{{\left(b_{1}^{n}\right)}^{2}}=1\\ ellipse2:\frac{{\left(x+c\right)}^{2}}{{\left(a_{2}^{m}\right)}^{2}}+\frac{{\left(y\right)}^{2}}{{\left(b_{2}^{m}\right)}^{2}}=1 \end{array}$$

here $a_{1}^{n}$, $b_{1}^{n}$, $a_{2}^{m}$ and $b_{2}^{m}$ represent the major and minor axes of the *n*th and *m*th ellipses respectively (refer to Figure 6). These terms are derived from Fresnel zone theory using the values of *m* and *n* and *c* is half the distance between the transmitter and receiver, as established during the system setup. By substituting the *x*, *y* values from Equations (2)–(3), we can determine the user’s direction $\theta_{p}$ and the LoS crossing point (ls,0).

### 4.3. Phone-Based Direction Estimation

**Figure 7:**
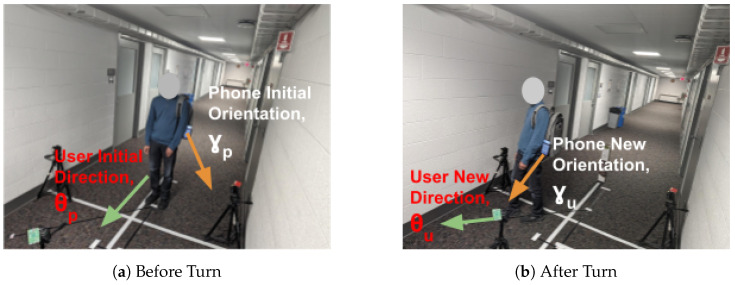
Mapping Phone Orientation to User Direction.

Different ways of user carrying the phone cause different misalignments between the phone’s orientation and the user’s walking direction. As long as the user doesn’t change the way they hold the phone, the mapping between the two directions doesn’t change. SWiLoc builds this mapping from $\theta_{p}$ and the phone’s orientation at location P, as shown by Equation (4).

(4) $$\theta_{u}=\left(\theta_{p}+\left(\gamma_{u}-\gamma_{p}\right)\right)\;\mathrm{mod}\;360$$

where $\gamma_{u}$ is the phone’s new orientation and $\theta_{u}$ is the user’s new direction. Figure 7 illustrates this mapping: user’s current direction obtained from correction zone is $\theta_{p}$ and phone’s current orientation is $\gamma_{p}$. After user takes a turn, user’s new direction $\theta_{u}$ is inferred by correcting phone’s new orientation $\gamma_{u}$ using the mapping in Equation (4). We calculate the phone’s orientation towards the global north (i.e., azimuth, which is the rotational angle about the phone’s z axis) by its 3D accelerator’s and magnetometer’s readings.

### 4.4. Location Estimation

With the accurate direction estimation, we can now accurately estimate the user’s location, that involves 3 main steps: step detection, step length estimation, and location calculation. By detecting each step, estimating the length of each step, and iteratively updating the position, we can continuously track the user’s movement and calculate their new location.

#### 4.4.1. Step Detection

To detect steps using smartphone accelerometer, we first compute the magnitude of the acceleration vector to combine the three axes and reduce noise using the Equation (5).

(5) $$a_{m}\left(t\right)=\sqrt{a_{x}{\left(t\right)}^{2}+a_{y}{\left(t\right)}^{2}+a_{z}{\left(t\right)}^{2}}$$

Next, we apply a low-pass filter to the magnitude signal to remove high-frequency noise, which has been done using the Equation (6), where α is the smoothing factor.

(6) $$a_{f}\left(t\right)=\alpha \cdot a_{m}\left(t\right)+\left(1-\alpha \right)\cdot a_{f}\left(t-1\right)$$

Finally, peak detection in the filtered signal is done by identifying local maxima that exceed a predefined threshold *T*, using the condition $a_{f}\left(t\right)>T$; and $a_{f}\left(t\right)>a_{f}\left(t-1\right)$ and $a_{f}\left(t\right)>a_{f}\left(t+1\right)$ to confirm a peak at time *t*. By applying these steps, we can accurately detect steps based on the identified peaks in the filtered acceleration data.

#### 4.4.2. Step Length Estimation

To estimate step length using the step detection algorithm, we collect accelerometer and gyroscope data from the smartphone during walking and identify steps using the filtered acceleration magnitude. For each detected step, we extract the mean acceleration magnitude $Acc_{mean}$, peak acceleration $Acc_{peak}$, mean gyroscope reading $Gyro_{mean}$ and step frequency $S_{freq}$ features using the Equation (7):

(7) $$\begin{array}{c} Acc_{mean}=\frac{1}{n}\sum_{i=1}^{n}a_{m}\left(i\right)\\ Acc_{peak}=max\left(a_{m}\left(i\right)\right)\\ Gyro_{mean}=\frac{1}{n}\sum_{i=1}^{n}\sqrt{\omega_{x}{\left(i\right)}^{2}+\omega_{y}{\left(i\right)}^{2}+\omega_{z}{\left(i\right)}^{2}}\\ S_{freq}=\frac{1}{\Delta t} \end{array}$$

These features are then combined into a feature vector *x* and given as follows:

(8) $$x={\left[\begin{array}{cccc} {\mathrm{Acc}}_{\mathrm{mean}} & {\mathrm{Acc}}_{\mathrm{peak}} & {\mathrm{Gyro}}_{\mathrm{mean}} & S_{\mathrm{freq}} \end{array}\right]}^{T}$$

The step length *L* is estimated using a dot product with a calibration vector *w*, such that L=w·x, where *x* is obtained from Equation (8). To determine the calibration vector *w* for accurate step length estimation in our system, the following procedure was implemented:

Initially, extensive sensor data were collected from controlled walking trials, ensuring precise measurement of actual step lengths. This data facilitated the extraction of key features, encapsulated in the vector *x*, which includes mean acceleration, peak acceleration, mean gyroscope reading, and step frequency, as outlined in Equation (7). A linear regression model was then applied to this dataset, correlating the feature vectors with the recorded step lengths to derive the coefficients forming *w*. This regression process was conducted using robust statistical software capable of linear regression analysis, allowing for the optimization of the calibration vector to ensure the most accurate and reliable step length predictions based on our sensor setup. This structured approach ensures that the step length estimation is finely tuned to the dynamics captured by our system’s sensors.

#### 4.4.3. Location Calculation

Given that the user, after reaching at point *P*, continues walking in the new direction $\theta_{u}$, then the new position $\left(x_{i},y_{i}\right)$ can be calculated iteratively using the following formulas [32]:

(9) $$\begin{array}{c} x_{i}=x_{i-1}+L_{i}\cdot \cos\left(\theta_{u}\cdot \pi /180\right)\\ y_{i}=y_{i-1}+L_{i}\cdot \sin\left(\theta_{u}\cdot \pi /180\right) \end{array}$$

where $x_{i-1}$ and $y_{i-1}$ are the coordinates of the previous position and $L_{i}$ is the estimated length of the *i*-th step. By updating the position after each detected step, we can continuously track the user’s location in the new direction $\theta_{u}$.

## 5. System Implementation

In this section, we outline the hardware configuration and the procedural steps required to implement the SWiLoc software.

### 5.1. Hardware Setup

Each correction zone consists of four receivers and one transmitter. We utilize the Raspberry Pi 4B as the receiver to gather CSI data, chosen for its accessibility and affordability. However, the standard firmware of the Broadcom WiFi chip does not support CSI data capture. Thus, we employed modified firmware [18] from Nexmon that enables CSI data capture. This firmware conveys CSI data to the host system by embedding it within transport layer payloads in frames. For transmission, a TP-Link Archer A7 router operating on the 5 GHz band was used. To initiate data transmission, we ping the router every 5 ms, prompting it to send a pong packet received by the four receivers.

We use a desktop equipped with an Intel Core i7 processor and 8 GB of RAM as the server, which connects to all receivers via Ethernet. This server processes the CSI data and performs all necessary calculations for direction and location computation. Additionally, it operates a Network Time Protocol (NTP) server to manage time synchronization across all system components.

Finally, a Huawei Nova 3i smartphone serves as the user’s device. It communicates with the server via a TCP socket and automatically synchronizes its time with the NTP server.

### 5.2. Software Implementation

We implement the following procedures on the server, with the android App installed on the phone:

#### 5.2.1. LoS Crossing Detection

**Figure 8:**
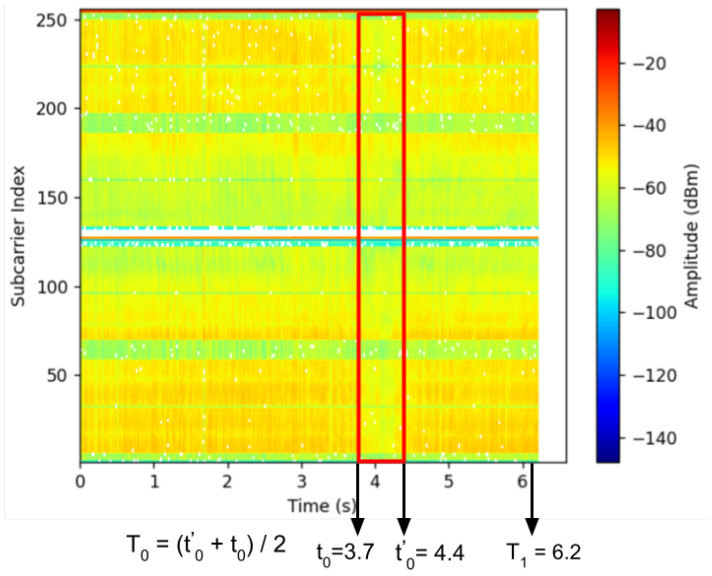
LoS Crossing Detection.

The LoS crossing detection step identifies a specific pattern in the CSI readings from four receivers and determines the time $T_{0}$ as the LoS crossing moment, along with the specific receiver (e.g., Rx1, 2, 3, or 4) where the user intersects the LoS. A distinct pattern emerges in the CSI data as the user crosses the LoS. Figure 8 displays this pattern (highlighted within a red rectangular box) occurring between $t_{0}=3.7$ s and $t_{0}^{\prime }=4.4$ s. The pattern reveals that the energy amplitude drops below −55 dBm during this interval. This reduction occurs because the user obstructs the radio signal between the transmitter and receiver, leading to decreased energy in the CSI readings. Given the brief duration of the LoS crossing (less than 1 s), we calculate $T_{0}$ as the midpoint of the interval, $T_{0}=\left(t_{0}+t_{0}^{\prime }\right)/2$, representing the average time of crossing.

**Figure 9:**
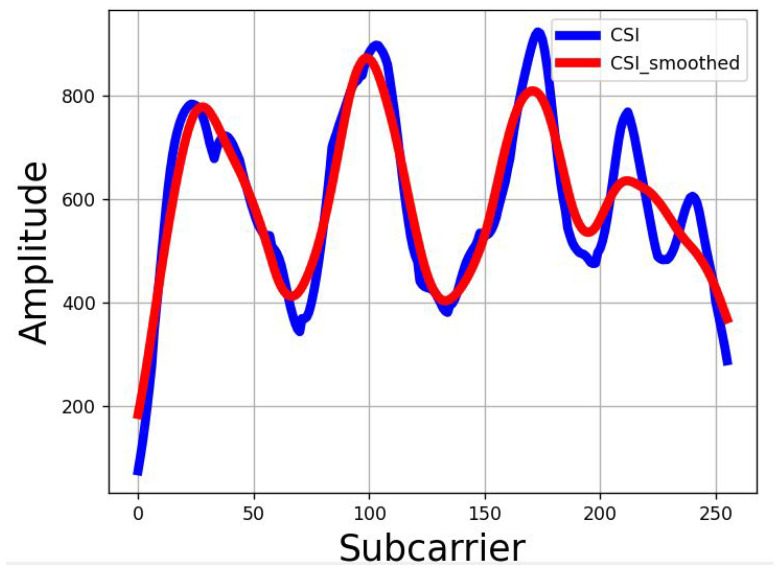
Before and After CSI Smoothing.

We developed a Python script that employs a simple search technique to identify the pattern. The script looks for consecutive amplitude values that meet or fall below a set threshold in the time domain. The size of the pattern window varies depending on the user’s body shape, walking speed, and the hardware specifications. Through repeated experiments, we observed that in our hardware and correction zone setup, a person of average size walking at a speed between 0.5 to 1 m/s can be accurately detected by setting a threshold amplitude value of −55 dBm. This parameter has been used for our evaluations. It is to be noted that the CSI data, as depicted in Figure 9, were captured before crossing the LoS during the 2nd to 3rd sec time interval shown in Figure 8, indicating a higher average raw amplitude values (>600).

#### 5.2.2. CSI Fluctuation Count

This phase determines *m* and *n* by counting fluctuations in the CSI data collected by Rx2 and Rx4 between $T_{0}$ and $T_{1}$, where $T_{0}$ is identified through LoS crossing detection and $T_{1}$ is provided by the smartphone. As a person moves along a radio propagation path, they create peaks and valleys in the CSI data within the Fresnel zone. By counting these peaks and valleys, we can ascertain which Fresnel zone the user occupies after walking *k* steps.

CSI data captured by the Raspberry Pi contains noise. To mitigate this, we employ the Least-square smoothing filter [33], which effectively smooths the CSI data while minimally altering the waveform. This filter creates a polynomial fit based on a predefined number of input samples, known as a sample window. After thorough testing across various environments, walking directions, and distances, we have empirically set the window size at 51. Figure 9 displays the CSI data before and after the smoothing process. We then utilize the *find_peaks* function from the *SciPy* package to count the fluctuations. Through experimental evaluation, we set the minimum height as 750 (measured in raw amplitude values, not dBm) to identify a fluctuation.

#### 5.2.3. Direction Calculation

Once *m* and *n* are determined, the subsequent step involves solving the two ellipse equations presented in Equation (3). For a given *n*, the boundary of the *n*-th Fresnel zone, $b_{1}^{n}$, formed by Rx2, is specified by the Fresnel zone model as follows:

(10) $$b_{1}^{n}=\{|Rx_{2}P|+|PTx|-|TxRx_{2}\left|=n\lambda /2\right\}$$

Here, λ represents a parameter set by the radio wavelength. Similarly, the boundary for the *m*-th Fresnel zone $b_{2}^{m}$, associated with Rx4, is also determined. Furthermore, for ellipse1 and ellipse2, three additional properties derived from Fresnel zone theory are essential for calculating $a_{1}^{n}$, $b_{1}^{n}$, $a_{2}^{m}$ and $b_{2}^{m}$ in Equation (3). For clarity, the three properties for ellipse1 are outlined below:

(11) $$\begin{array}{c} |Rx_{2}P|+|PTx|=2a_{1}^{n}\\ |TxRx_{2}|=2c\\ {\left(a_{n}^{1}\right)}^{2}-c^{2}={\left(b_{n}^{1}\right)}^{2} \end{array}$$

By solving Equations (10) and (11), we derive $b_{1}^{n}$ as follows:

(12) $$b_{1}^{n}=\frac{\sqrt{n\lambda^{2}+8n\lambda c}}{4}$$

Inserting $b_{1}^{n}$ into Equation (11), allows us to calculate $a_{1}^{n}$, with *c* as a predetermined variable (from Equation (3)). Similarly, the values for $a_{2}^{m}$ and $b_{2}^{m}$ for ellipse2 can be determined. This allows us to solve Equation (3) and determine the two unknown variables $\theta_{p}$ (direction) and ls, as illustrated in Figure 5b. With $\theta_{p}$ and ls distance established, the user’s location coordinate *x* and *y* at point *P* can readily be calculated using Equation (2).

#### 5.2.4. Location Calculation

Once we have determined the calculated direction ($\theta_{p}$), we apply Equation (4) to deduce the user’s new movement direction ($\theta_{u}$) based on changes in the phone’s orientation, which occur due to the user’s changed direction. Finally, we use Equation (9) to compute the user’s new location ($x_{i}$, $y_{i}$) by utilizing the updated direction ($\theta_{u}$).

#### 5.2.5. SWiLoc App Implementation

Our app development is based on an open-source compass application [34]. Specifically, our app (1) interacts with the server to obtain $T_{0}$ and $\theta_{p}$, and transmits $T_{1}$ and *d*; (2) detects the user’s steps and logs the time and the phone’s orientation at each step; (3) calculates the real-time direction of the user by Equation (4); (4) computes the successive locations of the user using the Equation (9).

## 6. Evaluation

This section outlines the procedure and findings of our assessment. We first measured the accuracy achieved by phase 1 only, and then further measured our overall accuracy, i.e., phases 1 and 2.

### 6.1. Testbed Setup

We carried out experiments in two distinct indoor settings to assess the performance of SWiLoc effectively. These included a lab office measuring 7.5 m by 6 m and a large empty corridor of 46 m by 3 m. In each environment, we positioned four receivers to create a 3 m by 3 m correction zone. The lab office, equipped with four tables, four chairs, and several desktops and monitors, is characterized by rich multi-path reflections. Conversely, the corridor is indicative of an environment with static reflections.

**Figure 10:**
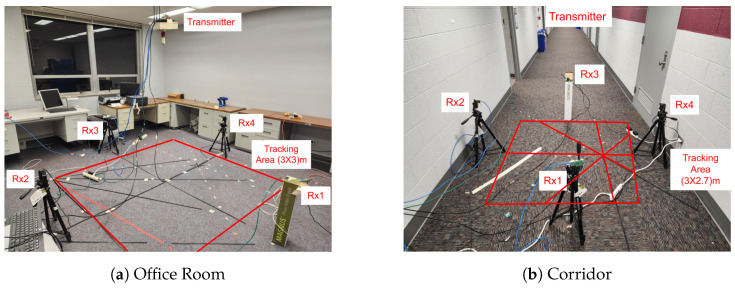
Testbed Setup.

The receivers, mounted on tripods 50 cm above the floor, are depicted in Figure 10. Each Raspberry Pi was positioned 1.5 m from the WiFi router, which was affixed to the ceiling directly above the midpoint of the four receivers. Keeping the receivers elevated from the ground is crucial to minimize the reflection of radio waves off the floor.

### 6.2. Performance Evaluation for Phase 1 Only

Primarily, we evaluated the WiFi sensing method used in phase 1 by testing the accuracy of direction estimation across eight basic paths. These paths intersected the WiFi router from eight different directions, each separated by 45 degrees: 0, 45, 90, 135, 180, 225, 270, and 315 degrees, relative to the line from Rx2 to Rx4. Since smartphone-based distance estimation is recognized for its accuracy and is not the primary focus of our study, we proceeded under the assumption that the distance *d* recorded by the smartphone was precise. Our experiment focused solely on measuring the errors originating from our WiFi sensing method. To accurately set the directions for each path, we employed a digital protractor and placed markers on the floor. Three volunteers were instructed to walk from a specific start point to an endpoint, ensuring their torsos were aligned with the marked line. We conducted eight repetitions for each data collection, collecting a total of 384 sets of WiFi CSI data across two environments, involving three volunteers, eight paths, and eight repeats each.

**Figure 11:**
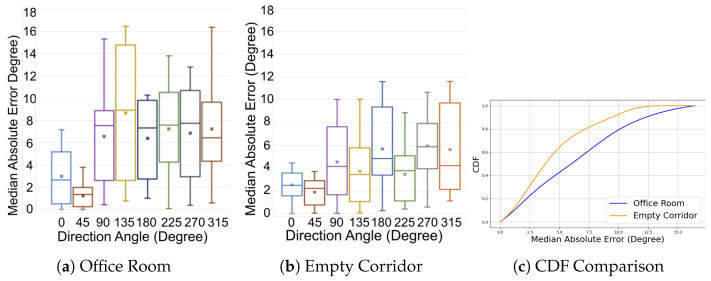
Phase 1 Accuracy in 2 Different Environments.

Figure 11 presents the Median Absolute Error (MAE) for eight fundamental paths within both an office room and an empty corridor. Figure 11a indicates that the overall MAE in the office room is 6 degrees, with a standard deviation of 5.41 degrees. Figure 11b displays that the overall MAE in the empty corridor is approximately 5 degrees, with a standard deviation of 4.04 degrees. Figure 11c illustrates that, in contrast to the office room, which is affected by substantial multi-path reflections, the empty corridor results in lower errors, with a 75th percentile error of about 6 degrees compared to 8 degrees in the office room. Notably, the 45-degree angle consistently exhibits less than 4 degrees of error in the upper quantile for both environments. The boxplots for both environments demonstrate that our approach accurately estimates unreliable directions (0, 90, 180 and 270 degrees).

### 6.3. Performance Evaluation for SWiLoc

We further incorporated a phone to evaluate SWiLoc’s overall accuracy (including both phase 1 and phase 2). Following our previous testing approach, we pre-defined 2 continuous traces (namely Trace 1 and 2) in corridor and demarcated the path on the ground to guide testers. Trace 1 and 2 have a length of 6.7m and 4.8m respectively. These continuous traces have multiple segments each, with some segments involving LoS crossing and other not. Five volunteers held the phone in 3 different positions, and the phone interacted with the server for direction estimation. To obtain the ground truth direction during walking, one observer used a laptop which was synchronized with the NTP server to record the time when testers started or took a turn. We repeated each data collection 5 times.

**Figure 12:**
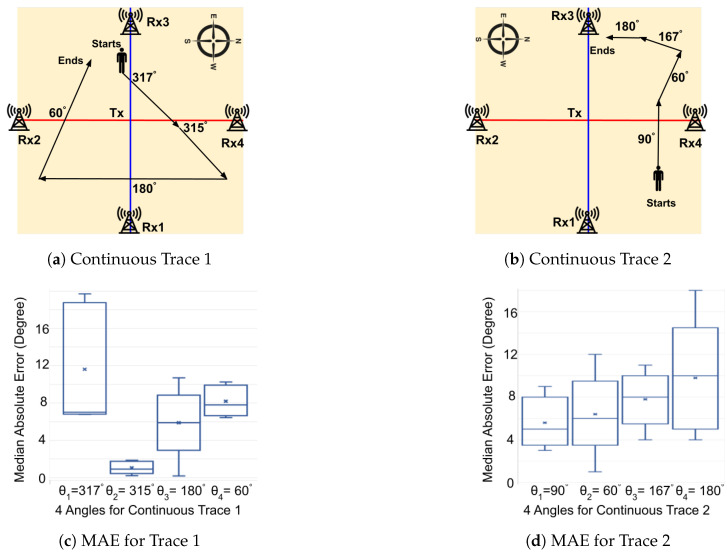
SWiLoc Performance in Continuous Traces.

Figure 12 illustrates the continuous traces and their corresponding accuracy for direction estimation. Trace 1 and trace 2 have 3 and 4 segments respectively. After each segment of walk, our tester changed their directions and the phone inferred their new directions based on the mapping obtained from phase 1, till the tester crossed another LoS. We observed that segment 2 exhibited higher accuracy than other segments in trace 1.

**Figure 13:**
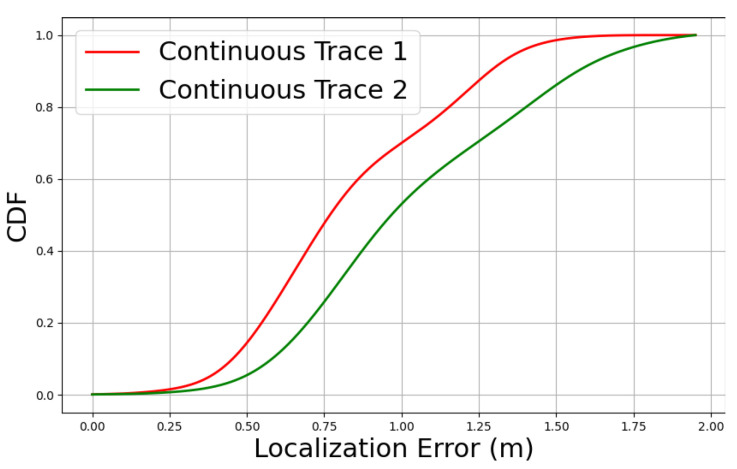
Localization Error for Continuous Trace 1 & 2.

We then infer the tester’s location with every 1 interval using the estimated walking direction of the tester. As mentioned previously, 5 volunteers walked through these 2 traces with an average speed of 0.63 m/s, with minimum and maximum speed of 0.51 m/s and 0.96 m/s respectively. Therefore, our system calculated approximately 300 locations (around 12 locations × 5 volunteers × 5 repetition) for trace 1 and 200 locations (around 8 locations × 5 volunteers × 5 repetition) for trace 2. Figure 13 shows the Cumulative Distribution Function (CDF) for localization error for both of the traces. Trace 1 exhibits less error than trace 2, as trace 1 consists of 4 LoS crossing points. On the other hand, trace 2 has only 1 LoS crossing point.

**Figure 14:**
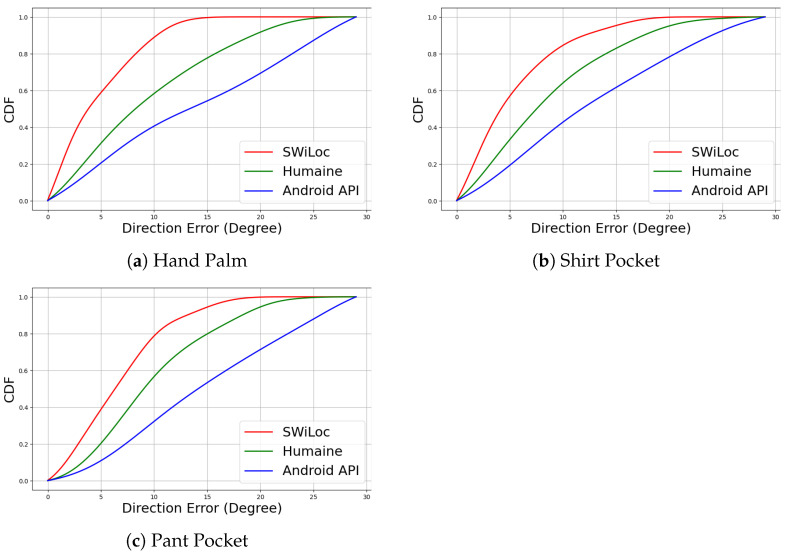
CDF of Different Phone Holding Positions, Comparing SWiLoc with Humaine and Android API.

We also tested how different ways of holding the phone impacted the walking direction accuracy. Figure 14a–c shows the CDF graphs for 3 hold positions, comparing SWiLoc with two other systems, Humaine [7] and Android’s builtin compass. The CDF results show that SWiLoc achieves the best accuracy regardless of the phone holding position. These results provide evidence to support our claim that combining active smartphone-based PDR with passive WiFi-sensing-based correction zones yields greater accuracy than existing state-of-the-art systems.

In addition to that, we computed the median and 75th percentile accuracy achieved by SWiLoc across all test scenarios and compared these figures with three other state-of-the-art works: WiDir [28], Widar [29] and WalkCompass [23]. WiDir and Widar utilize passive WiFi sensing, whereas WalkCompass relies on smartphone technology. We derived the accuracies of these methods from their respective publications, as we did not have access to their source codes. As detailed in Table 1, SWiLoc surpasses the performance of these models in terms of 75th percentile and median errors, although Widar records a slightly lower median error of 5 degrees compared to SWiLoc’s 6 degrees. Against WalkCompass, the closest competitor, SWiLoc achieves a 64% reduction in the 75th percentile error. This superior performance is due to the use of a geometrical relationship that avoids the cumulative errors commonly seen in other models.

**Figure 15:**
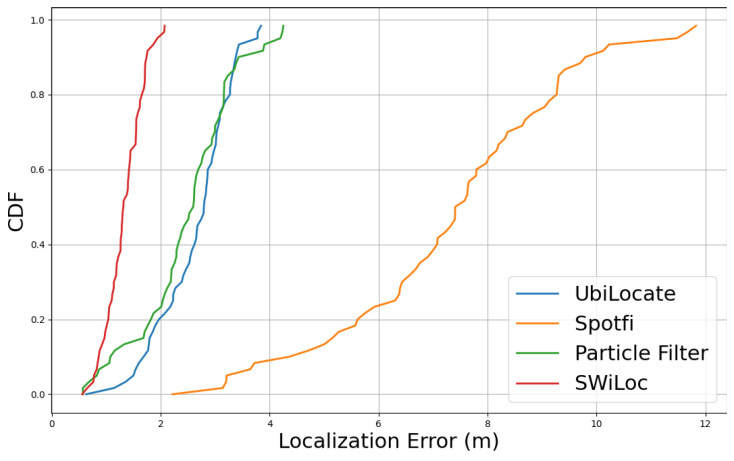
Localization Error for Different State-of-the-art methods (Holding Phone on Hand Palm).

Finally, we compared our localization results with the state-of-the-arts localization approaches, shown in Table 2. Among the approaches, UbiLocate [35], Spotfi [36], Spring [37], Fusic [38] are based on passive sensing, while Kalman-filter [39], SmartPDR [24], Particle-filter based [40], LSTMLoc [41] are active fusion based. SWiLoc outperformed both the active and passive based localization approaches by achieving 1.12 m of 80-th percentile localization error. Among all the methods, we implemented UbiLocate, Spotfi and Particle Filter based methods and tested for only Trace 2 holding the phone on hand palm. Figure 15 demonstrated that SWiLoc achieved highest localization accuracy among the 3 other methods. Please note that we employed Intel 5300 NIC for implementing Spotfi and UbiLocate testbed and utilized Linux CSI tool [42] to collect and process CSI data.

### 6.4. Sensitivity Analysis of SWiLoc

In this subsection, we analyzed the sensitivity of SWiLoc by examining how varying LoS crossing locations, distances and direction accuracy impact overall system performance, particularly focusing on direction and localization accuracy.

#### 6.4.1. Impact of Varying LoS Crossing Locations

**Figure 16:**
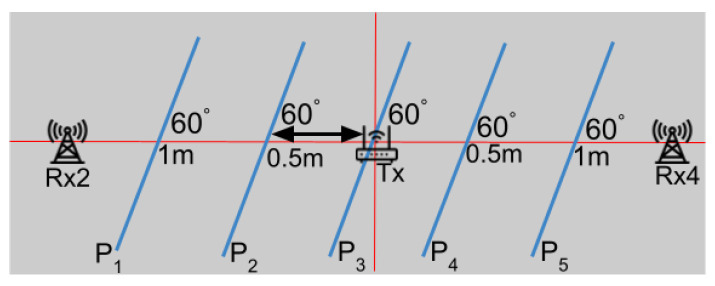
Paths with Different LoS Crossing Locations.

**Figure 17:**
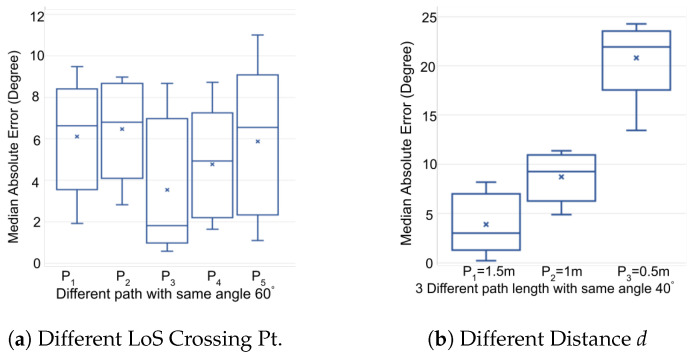
Impact of LoS Crossing Locations and Distances.

We evaluated the impact of varying LoS crossing locations on accuracy. As depicted in Figure 16, the testers traversed the LoS between Rx2 and Rx4 maintaining a consistent walking direction, but at different crossing points. These paths ($P_{1}$, $P_{2}$, $P_{3}$, $P_{4}$ and $P_{5}$), each 3 m in length and spaced 0.5 m apart, are further analyzed in Figure 17a. The results show that the direction estimation for all paths exhibited a median error of less than 7 degrees, demonstrating that our WiFi sensing method performs well across various LoS crossing points.

#### 6.4.2. Impact of Distance *d* on Direction Accuracy

We noticed that the influence of the human body on accuracy is significant. While our models treat the human body as a point, the actual width of the body impacts the CSI reflections and Fresnel zone fluctuation counts. This effect diminishes if the testers walk a longer distance post-LoS crossing. Consequently, we conducted further experiments to assess how the distance *d* (the path length after crossing the LoS) affects accuracy. The testers crossed the LoS maintaining a constant walking direction but varying walking distances *d* (i.e., 0.5 m, 1 m, 1.5 m). Figure 17b shows that shorter distances *d* lead to greater errors. From these findings, it is advisable for developers setting up correction zones to ensure that area allows users to walk at least 0.5 m after crossing the LoS to minimize errors.

#### 6.4.3. Impact of Direction Accuracy on Localization Accuracy

**Figure 18:**
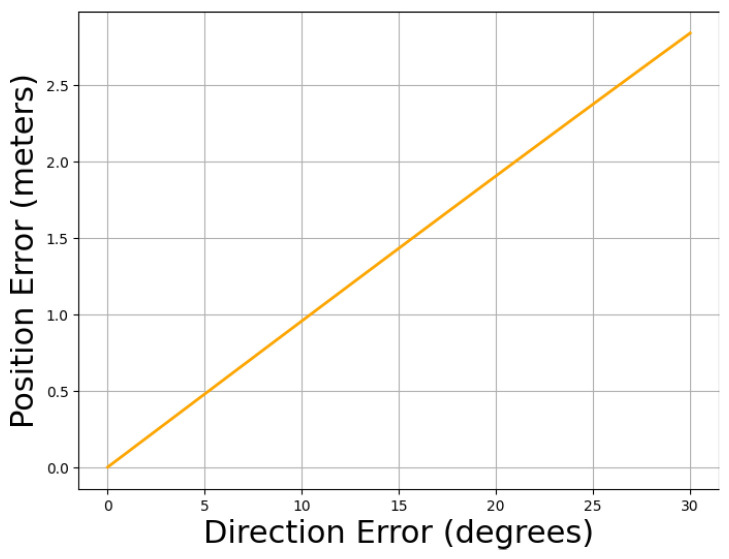
Impact of Direction Error on Loc. Accuracy.

While it is evident that greater directional error leads to increased localization inaccuracies, quantifying this error propagation is crucial. Therefore, we have systematically measured how errors in direction influence localization accuracy. Figure 18 shows that as direction errors increase, the position error grows non-linearly, indicating that precise direction estimation is crucial. Moderate to large direction errors, like 30 degrees, lead to substantial deviations in the final location, emphasizing the importance of minimizing direction errors to maintain accurate localization.

### 6.5. Discussion

To the best of our knowledge, SWiLoc represents the first attempt to merge passive WiFi sensing with smartphone-based direction estimation. Our evaluations suggest that SWiLoc delivers robust accuracy compared to other cutting-edge models. Additionally, it requires no training, enabling it to be deployed in a plug-and-play manner.

We also notice some of its limitations as well as special consideration for deployment, as stated below:

(1) **Requirements on Physical Deployments:** Similar to other RF-based human sensing applications like patient monitoring and gesture recognition, SWiLoc is affected by complex multi-path environments, and its accuracy significantly decreases when two individuals enter the same correction zone at the same time. Additionally, it is necessary for the pedestrian to walk at least 0.5 m after crossing the LoS. Therefore, when implementing SWiLoc, developers must ensure these conditions are met to maintain system effectiveness.
(2) **Assumption on Phone’s Orientation:** Phase 2 of SWiLoc relies on the assumption that the mapping between user’s direction and phone’s orientation won’t change. However, sometimes a pedestrian may change how they carry the phone. Considering this, it is necessary to deploy multiple correction zones to update the mapping. Besides, as a future work direction, we plan to integrate our approach with other sensor fusion-based approaches such as WalkCompass.
(3) **Configuration of Correction Zone:** Configuring correction zones effectively is crucial for ensuring reliable and accurate localization. The coverage area of each correction zone is primarily determined by the sensing capabilities of each locations and the distance between the deployed transceivers. Based on recent advancements in CSI based sensing, as noted in the latest literature, transceivers can maintain effective sensing zone over distances up to 40 m [43]. This extended range allows us to design larger correction zones, thereby reducing the number of zones required to cover a given space comprehensively.
  To this end, to determine the optimal number and placement of correction zones, we can employ a systematic approach that considers the layout of the indoor environment and the typical movement patterns of users within it. The placement strategy aims to maximize coverage, ensuring that at any point within the environment, a pedestrian’s mobile phone can reliably connect to at least one correction zone. This is particularly important given the random nature of changes in phone posture and orientation as users move.
(4) **Applicability of SWiLoc in Diverse Indoor Environments:** Our SWiLoc system employs a seamless integration with server-based corrections, which are delivered to users via an existing WiFi connection. Users are not required to be aware of the server or the technicalities of correction zones; instead, they experience an automated and continuous improvement in direction and location accuracy as they move within the coverage area. This setup ensures that the localization system is user-friendly and unobtrusive, leveraging WiFi connectivity to provide necessary updates and corrections from the server. This design is particularly effective in environments where WiFi is readily available, allowing for broad application in various indoor settings such as shopping centers, offices, hospitals, airports, museums etc. without the need for specialized knowledge or interaction from the user.

In practical, to enhance this seamless navigation experience, our system utilizes multiple correction zones distributed throughout the indoor space. As the user moves and remains connected to the WiFi network, the system automatically detects which correction zone the user is currently in. Based on this location information, the corresponding correction data from the nearest or most relevant correction zone is dynamically provided to the user. This ensures that the direction corrections are contextually accurate, enhancing the reliability of the navigation solution as the user traverses different areas within the space. This dynamic allocation of correction data based on user’s real-time location within the correction zones significantly improves the overall system efficacy and user experience in navigating complex indoor environments.

## 7. Conclusions

In this paper, we introduce SWiLoc, a system designed to precisely estimate movement direction and location by integrating passive WiFi sensing with smartphone-based direction estimation. Our innovative approach incorporates extra receivers to enhance WiFi sensing and employs a geometric method to improve direction and thereby localization accuracy. SWiLoc, implemented using Raspberry Pi, demonstrates through comprehensive testing an average 80th percentile error of 1.12 m in location estimation, achieving a 49% reduction over existing state-of-the-art methods.

## Figures and Tables

**Table 1:** Comparing SWiLoc’s Walking Direction Accuracy (Phase 1) with Other Methods.

Approaches	75th Percentile Error (in degree)	Median Error (in degree)
WiDir	23	10
WalkCompass	14.2	8
WiDar	18	5
**SWiLoc (Phase 1)**	**8.89**	**6**

**Table 2:** Comparing SWiLoc’s Localization Accuracy (Phase 1 & 2) with Other Methods.

Approaches	80th Percentile Localization Error (m)	Base Sensing Method
UbiLocate	2.2	Passive
Spotfi	6.0	Passive
Spring	3.7	Passive
Fusic	3.4	Passive
Kalman-filter based	4.6	Active Fusion
PDRLoc	6.71	Active Fusion
Particle-filter based	2.21	Active Fusion
LSTMLoc	2.36	Active Fusion (Training-based)
**SWiLoc (Phase 1 & 2)**	**1.12**	**Active Fusion & Passive, Training free**

## Data Availability

The original contributions presented in the study are included in the article, further inquiries can be directed to the corresponding author.
